# Psychological Vulnerability During Pregnancy and Its Obstetric Consequences: A Multidimensional Approach

**DOI:** 10.3390/healthcare13172211

**Published:** 2025-09-04

**Authors:** Ioana Denisa Socol, Ahmed Abu-Awwad, Flavius George Socol, Simona Sorina Farcaș, Simona-Alina Abu-Awwad, Bogdan-Ionel Dumitriu, Alina-Iasmina Dumitriu, Daniela Iacob, Daniela-Violeta Vasile, Nicoleta Ioana Andreescu

**Affiliations:** 1 Doctoral School, University of Medicine and Pharmacy “Victor Babeş”, Eftimie Murgu Sq. No. 2, 300041 Timisoara, Romania; ioana.socol@umft.ro (I.D.S.); bogdan.dumitriu@umft.ro (B.-I.D.); alina.moatar@umft.ro (A.-I.D.); 2 Department XV-Discipline of Orthopedics-Traumatology, University of Medicine and Pharmacy “Victor Babeş”, Eftimie Murgu Sq. No. 2, 300041 Timisoara, Romania; ahm.abuawwad@umft.ro; 3 Research Center University Professor Doctor Teodor Sora, University of Medicine and Pharmacy “Victor Babeş”, Eftimie Murgu Sq. No. 2, 300041 Timisoara, Romania; 4 1st Clinic of Obstetrics and Gynecology, “Pius Brinzeu” County Clinical Emergency Hospital, 300723 Timisoara, Romania; 5 Department of Obstetrics and Gynecology, Faculty of Medicine, University of Medicine and Pharmacy “Victor Babeş”, Eftimie Murgu Sq. No. 2, 300041 Timisoara, Romania; 6 Department of Microscopic Morphology-Genetics, Center of Genomic Medicine, University of Medicine and Pharmacy “Victor Babeş”, Eftimie Murgu Sq. No. 2, 300041 Timisoara, Romania; farcas.simona@umft.ro (S.S.F.); andreescu.nicoleta@umft.ro (N.I.A.); 7 Department XII-Obstetrics-Gynecology, Discipline of Neonatology and Childcare, University of Medicine and Pharmacy “Victor Babeş”, Eftimie Murgu Sq. No. 2, 300041 Timisoara, Romania; daniela.iacob@umft.ro; 8 Faculty of Medicine, University of Medicine and Pharmacy “Victor Babeş”, Eftimie Murgu Sq. No. 2, 300041 Timisoara, Romania; daniela-violeta.vasile@student.umft.ro

**Keywords:** pregnancy complications, premature birth, low birth weight, anxiety disorders, depression, stress, psychological, adaptation, psychological

## Abstract

**Background/Objectives**: Maternal depression, anxiety, perceived stress, and resilience are recognized determinants of perinatal health, yet routine psychological screening is still uncommon in Romanian obstetric practice. This study examined how these four psychological factors relate to preterm birth, gestational hypertension, intra-uterine growth restriction (IUGR), and low birth weight in primiparous women. **Methods**: In a cross-sectional study at a tertiary maternity center in Timișoara (February 2024–February 2025), 240 women at 20–28 weeks’ gestation completed the Edinburgh Postnatal Depression Scale (EPDS), Generalized Anxiety Disorder-7 (GAD-7), Perceived Stress Scale-10 (PSS-10), and Connor–Davidson Resilience Scale-25 (CD-RISC-25). Obstetric outcomes were abstracted from medical records. Pearson correlations described bivariate associations; multivariate logistic regression assessed independent effects after mutual adjustment. **Results**: Preterm birth occurred in 21% of pregnancies, gestational hypertension in 17%, IUGR in 15%, and low birth weight in 21%. Higher EPDS, GAD-7, and PSS-10 scores correlated positively with each complication (r = 0.19–0.36; *p* < 0.02), whereas CD-RISC-25 scores showed inverse correlations (r = −0.22 to −0.29; *p* ≤ 0.012). In the fully adjusted model, GAD-7 remained the only independent psychological predictor of the composite obstetric outcome (β = 0.047; 95% CI 0.010–0.083; *p* = 0.013). Perceived stress approached significance; depression and resilience were no longer significant after adjustment. **Conclusions**: Generalized anxiety was the most robust psychological determinant of adverse obstetric outcomes, with perceived stress, depression, and lower resilience showing contributory roles at the unadjusted level. Incorporating brief instruments such as the GAD-7, PSS-10, and CD-RISC-25 into routine prenatal care could facilitate early identification of at-risk pregnancies and inform targeted preventive interventions.

## 1. Introduction

Preparing for motherhood is often viewed as a time of joy, anticipation, and personal transformation; however, pregnancy can also represent a period of heightened psychological vulnerability, due to a unique interplay of emotional, physical, and social stressors [1,2]. These challenges, ranging from uncertainty about the baby’s health to profound changes in personal identity and social roles, can significantly affect a woman’s mental well-being [3]. In recent years, a growing body of research has explored the impact of maternal psychological stress on adverse pregnancy outcomes [4]. Nevertheless, in routine clinical practice, these factors are rarely assessed or systematically addressed. Although recognized in clinical guidelines, maternal mental health screening remains infrequent in obstetric care. Recent U.S. data show that only about 5% of prenatal visits include documented screening, increasing modestly to 9% postpartum [5].

Emerging evidence indicates that maternal stress, anxiety, and depression during pregnancy are associated with an increased risk of complications such as preterm birth, low birth weight, intrauterine growth restriction (IUGR), and gestational hypertension [6,7]. The mechanisms involved are multifactorial and complex, including dysregulation of the hypothalamic–pituitary–adrenal (HPA) axis, excessive cortisol exposure, and inflammatory responses that may impair placental function and fetal development [8]. However, not all women exposed to elevated stress levels during pregnancy experience such outcomes, suggesting the existence of protective psychological factors, among which resilience is particularly noteworthy [9,10].

Psychological resilience, defined as the capacity to adapt and recover in the face of adversity, may serve as a crucial buffer mitigating the deleterious effects of stress on maternal and fetal health [11]. Despite its significant clinical relevance, resilience remains insufficiently explored in obstetrics, with standardized assessment tools rarely incorporated into routine prenatal care [12,13]. Similarly, although antepartum depression and anxiety are well-known risk factors for adverse outcomes, validated tools like the EPDS and GAD-7 are still inconsistently incorporated into prenatal screening protocols [14,15]. The same is true for the Perceived Stress Scale (PSS) and the Connor–Davidson Resilience Scale (CD-RISC), both of which can provide valuable insights into a pregnant woman’s coping capacity [16].

Although international guidelines support perinatal mental health screening, such practices remain uncommon in Romanian obstetric care, where psychological evaluation is often limited to severe cases or omitted altogether.

Given these contextual realities, the current research seeks to examine the relationship between psychological distress (depression, anxiety, perceived stress) and resilience in pregnant women, and to correlate these factors with key obstetric and fetal outcomes—specifically preterm birth, gestational hypertension, IUGR, and low birth weight. The ultimate goal is to assess whether psychological resilience can moderate the effects of maternal stress and whether a psychological risk profile can be outlined to improve early screening and individualized care during pregnancy, especially in times of collective adversity.

## 2. Materials and Methods

### 2.1. Study Design and Population

This study was conducted as an observational, cross-sectional investigation with an analytical component, within the Obstetrics and Gynecology Department of the “Pius Brînzeu” County Emergency Clinical Hospital in Timișoara (SCJUPBT).

Participant recruitment occurred between 1 February 2024 and 1 February 2025, during routine outpatient consultations. Eligible pregnant women were enrolled between 20 and 28 weeks of gestation—a period generally regarded as clinically and psychologically stable.

No a priori sample size calculation was performed, as the study was designed as an exploratory cross-sectional analysis. The final sample size of 240 reflects all eligible women consecutively recruited over a 12-month period at our tertiary maternity center.

Recruitment was consecutive from routine outpatient visits, not selective based on obstetric risk.

SCJUPBT, a level III medical center and university-affiliated hospital, serves an extensive geographic region in southwestern Romania. Consequently, participants were drawn from the counties of Timiș, Arad, Caraș-Severin, Hunedoara, and Mehedinți, ensuring a study sample with substantial socio-economic and geographic diversity, and thus strong regional representativeness.

Inclusion Criteria:

- Primiparous women, aged between 18 and 45 years.
- Women who are either married or in a stable partnership.
- Continuous employment for at least 24 months prior to conception.
- Minimum educational attainment of secondary (high school) level.
- A single, ongoing pregnancy with a gestational age between 20 and 28 weeks.
- Ongoing prenatal care, including clinical and ultrasound monitoring, provided by a gynecologist.
- Absence of obstetric complications or hospital admissions during the current pregnancy at the time of questionnaire administration.
- Adequate cognitive and linguistic abilities to complete self-reported questionnaires.
- Pregnant women who provided written informed consent, agreed to participate in follow-up until delivery, and supplied up-to-date contact information were included in the study.
- Documented record of hospitalizations or major medical events during the current pregnancy up to the point of evaluation.

Exclusion Criteria:

- Pre-existing psychiatric diagnoses prior to conception, current antenatal psychotropic treatment, recent psychiatric hospitalization or intensive psychiatric care, or active suicidal ideation/self-harm risk.
- Presence of significant chronic comorbidities.
- Use of alcohol or illicit drugs during pregnancy.
- Women with an active COVID-19 infection or hospitalization due to COVID-19-related complications in the three years prior to conception were excluded.
- Pregnancy achieved through assisted reproductive technologies.
- History of adverse events in previous pregnancies.
- Chronic use of corticosteroids or other immunomodulatory medications.
- Diagnosis of a severe acute infection at the time of assessment.
- Ongoing or recently documented cases of domestic violence.
- Homelessness or severely disadvantaged social circumstances.
- Placenta praevia or other known placental abnormalities at the time of inclusion.
- Major fetal anomalies detected via ultrasound or genetic testing.

### 2.2. Variables and Data Collection

In this study, data collection was carefully structured to enable a comprehensive assessment of both psychological and obstetric variables, aiming to identify potential associations between maternal mental health and fetal outcomes. All data were collected during a single outpatient visit occurring between 20 and 28 weeks of gestation—a period deliberately selected for its relative physiological and psychological stability.

Psychological variables

Participants completed four standardized, validated self-report instruments under the supervision of trained research personnel:

- Edinburgh Postnatal Depression Scale (EPDS): A 10-item screening tool for symptoms of antenatal and postnatal depression. A score of ≥13 was used as the clinical threshold indicating elevated depressive symptoms [17].
- Generalized Anxiety Disorder-7 item scale (GAD-7): This brief, 7-item instrument screens the frequency of core generalized-anxiety symptoms over the previous two weeks (scores range 0–21), with totals ≥10 indicating clinically significant anxiety that warrants further evaluation [18].
- Perceived Stress Scale (PSS): A 10-item scale assessing the degree to which participants appraise situations in their lives as stressful, with a threshold score of ≥20 suggestive of high perceived stress [19].
- Connor–Davidson Resilience Scale (CD-RISC): This 25-item instrument evaluates psychological resilience—defined as the capacity to cope with adversity. Higher scores denote stronger resilience, with no rigid clinical cut-off but population-based norms serving as reference points [16].

All questionnaires were administered in their validated Romanian translations and completed in a quiet, private setting within the outpatient unit. Clarifications were offered as needed, but participants were encouraged to respond independently and honestly.

Obstetric and Clinical variables

Maternal, medical, and obstetric data were extracted from prenatal records and updated at the time of questionnaire completion, as neonatal information was not yet available. The primary obstetric outcomes of interest were as follows:

- Preterm birth, defined as delivery before 37 completed weeks of gestation [20];
- Gestational hypertension, diagnosed according to current international criteria (new-onset hypertension after 20 weeks’ gestation in the absence of proteinuria or other systemic features) [21];
- Intrauterine growth restriction (IUGR), based on ultrasound-estimated fetal weight below the 10th percentile for gestational age [22];
- Low birth weight, defined as neonatal weight below 2500 g at birth [23].

Additional clinical variables collected were as follows: maternal age, body mass index (BMI), educational attainment, marital/partnership status, and place of residence (urban vs. rural). These variables were collected through structured interviews and confirmed via medical records where applicable.

To ensure data accuracy, all records were reviewed independently by two members of the research team. Discrepancies were resolved by consensus or in consultation with the supervising obstetrician. Each participant was assigned a unique anonymized code to ensure confidentiality throughout data handling and analysis.

This integrated approach allowed for the exploration of both vulnerability and protective psychological traits, and their potential impact on perinatal outcomes.

The study was conducted in accordance with the ethical standards of the institutional and national research committees, and with the 1964 Helsinki Declaration and its later amendments. Ethical approval was obtained from the Ethics Committee of the Timișoara County Emergency Clinical Hospital, under approval number 17/31 January 2024. All participants provided written informed consent prior to their inclusion in the study.

### 2.3. Statistical Analysis

Statistical analysis was performed using GraphPad Prism version 9.0 (GraphPad Software, San Diego, CA, USA). Continuous variables were presented as means ± standard deviations (SD), while categorical variables were reported as absolute frequencies and percentages.

To explore associations between psychological variables and obstetric outcomes, we conducted bivariate correlation analyses using Pearson’s correlation coefficient (r). This allowed us to evaluate the linear relationships between psychological scores (EPDS, GAD-7, PSS, CD-RISC) and key clinical outcomes, such as preterm birth, gestational hypertension, intrauterine growth restriction (IUGR), and low birth weight.

Subsequently, a multivariate logistic regression model was employed to examine the predictive value of each psychological variable while adjusting for potential confounding effects and shared variance. Regression coefficients, standard errors, z-scores, *p*-values, and 95% confidence intervals (CI) were reported for each predictor. A *p*-value < 0.05 was considered statistically significant.

All statistical tests were two-tailed, and the threshold for clinical relevance was evaluated in parallel with statistical significance, particularly in light of possible multicollinearity among psychological predictors.

## 3. Results

**Figure 1:**
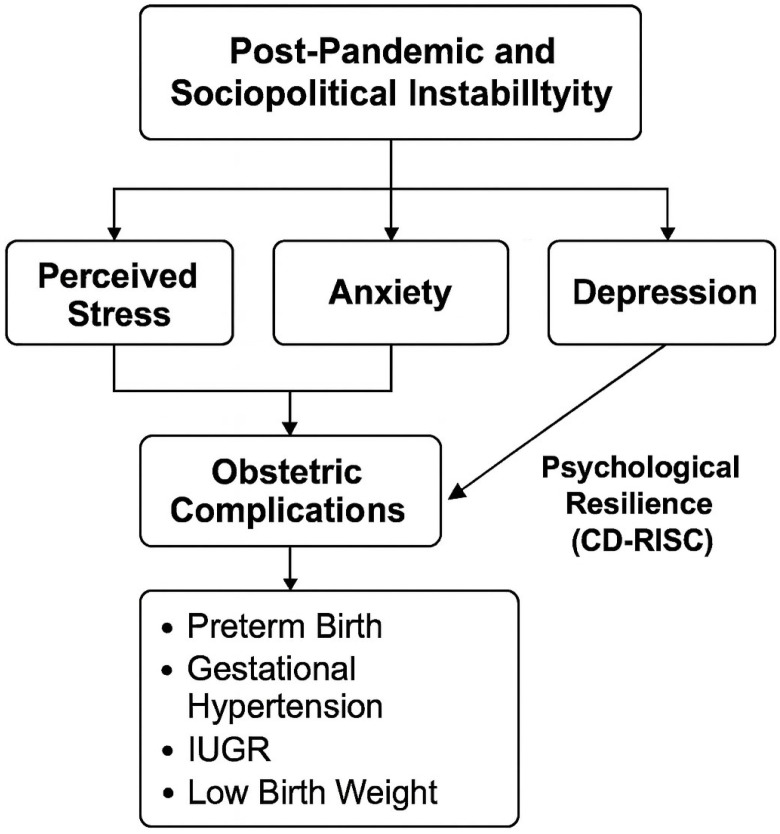
Conceptual model of the relationship between socio-political instability, maternal psychological factors, and obstetric outcomes.

The conceptual diagram illustrates the hypothesized pathways through which post-pandemic and socio-political instability may contribute to adverse obstetric outcomes, mediated by elevated levels of perceived stress, anxiety, and depression during pregnancy. Psychological resilience is depicted as a moderating variable, potentially buffering the negative effects of psychological distress on maternal and fetal health. This model reinforces the rationale for integrating mental health screening into routine prenatal care, particularly during periods of widespread societal stress (see Figure 1).

A cohort of 240 pregnant women, enrolled between 20- and 28-weeks’ gestation, constituted the final analytic sample. Mean gestational age at delivery reached 39 weeks, indicating that nearly all births occurred at term. With a mean pre-pregnancy BMI of 25.74, the group fell within the normal-weight range. Educational attainment was notably high: the majority had completed tertiary studies, while the remainder held secondary-level qualifications. Participants were predominantly urban residents and were largely married or in stable partnerships. Collectively, these attributes depict a relatively homogeneous, middle-class population with reliable access to routine prenatal care—traits that provide essential context for interpreting the psychological and obstetric outcomes reported in the study. The full set of sociodemographic and clinical descriptors can be found in Table 1.

The cohort was relatively homogeneous in terms of age, BMI, marital/partnership status, and educational attainment, with no marked demographic or clinical imbalances across psychological subgroups.

The psychometric profile of the cohort sketches a complex landscape of antenatal mental health (Table 2). Depressive symptoms, gauged with the EPDS, cluster in the mild-to-moderate range, yet a sizeable minority already meet clinical criteria—an early signal that mood surveillance should not wait until the postpartum period. Anxiety follows a parallel, slightly steeper trajectory: the GAD-7 indicates moderate symptom severity on average, and roughly one woman in two scores above the cut-off, underscoring the pervasiveness of generalized-anxiety features in pregnancy. Self-reported stress paints an even more vivid picture, with a clear majority of respondents endorsing levels consistent with at least moderate perceived stress, a pattern that dovetails with the elevated depression and anxiety indices.

Resilience, assessed by the CD-RISC, offers a partial counterweight: most participants fall within the mid-range of adaptive capacity, suggesting access to some coping resources. Even so, nearly half of the registered scores are commonly interpreted as low resilience, hinting that protective factors may be insufficient for a substantial subset. In concert, these findings portray a population in which heightened depressive mood, anxiety, and stress converge against a backdrop of only moderate resilience—an interplay that argues for integrated, preventative psychological interventions during routine prenatal care.

The obstetric complications observed in this cohort delineate a morbidity profile that warrants close clinical attention. Preterm delivery was recorded in approximately one out of five pregnancies, underscoring a perinatal vulnerability that may be amplified by both biological and psychosocial stressors. Gestational hypertension was identified in nearly one in six participants, highlighting the need for early blood pressure monitoring and timely nutritional or pharmacological interventions. The incidence of intrauterine growth restriction parallels that of hypertensive disorders, while low birth weight nearly overlaps with the preterm birth rate, an alignment that foreshadows immediate neonatal challenges. Collectively, these observations depict an obstetric population exposed to substantial risk and argue for integrated antenatal monitoring that aligns clinical indicators with the psychological markers discussed earlier. A detailed breakdown of each complication appears in Table 3.

The correlation matrix sharpens the picture painted by the raw prevalence data. Depressive mood, indexed by the EPDS, shows small-to-moderate positive associations with every obstetric complication examined—preterm birth, gestational hypertension, intra-uterine growth restriction (IUGR), and low birth weight—each relationship reaching conventional levels of statistical significance. Anxiety, as assessed by the GAD-7, emerged as an even more consistent predictor. Correlation coefficients ranged from the mid-0.20 s to low-0.30 s, suggesting that generalized anxiety symptoms contribute an additional and distinct layer of risk beyond that associated with depressive symptoms alone.

Perceived stress emerged as the strongest psychological correlate. The PSS displayed the largest positive associations across all obstetric outcomes, indicating that a pregnant woman’s subjective appraisal of stress may be the most immediate and influential driver of obstetric vulnerability within this cohort. In contrast, psychological resilience—as measured by the CD-RISC—showed inverse but moderate correlations with the same complications. This finding supports the notion that adaptive capacity may buffer, though not entirely neutralize, the adverse impact of negative emotional states during pregnancy.

Taken together, these findings extend the descriptive patterns reported earlier: higher burdens of depression, anxiety and perceived stress align with clinically meaningful elevations in adverse obstetric outcomes, whereas greater resilience appears modestly protective. Although cross-sectional data cannot establish causality, the results highlight a consistent and converging pattern: maternal psychological well-being is intrinsically linked to perinatal physiology—an association that epidemiological research can no longer afford to ignore. Full statistical details are provided in Table 4.

Although all participants met low-risk criteria at inclusion, the observed rates of complications are higher than national averages, reflecting the specific patient population of a tertiary referral center and the broader post-pandemic socio-political context.

In the multivariable model, generalized anxiety (GAD-7) emerged as the only independent psychological predictor, maintaining a statistically significant association with adverse obstetric outcomes even after controlling for depressive symptoms (EPDS), perceived stress (PSS), and psychological resilience (CD-RISC). The coefficients for depression and perceived stress remained positive, while that for resilience stayed negative, yet none of these reached statistical significance once anxiety was accounted for. These findings underscore the distinctive weight of antenatal anxiety in shaping obstetric risk, as detailed in Table 5 below.

## 4. Discussion

Building on our findings, we advocate for the development and validation of a structured psychological risk profile tailored to pregnancy. This tool would facilitate early identification of women vulnerable to obstetric complications linked to psychological distress. It should integrate key psychological indicators, such as perceived stress, depressive and anxiety symptoms, and resilience capacity, alongside contextual variables like family support and socio-economic status. Such a profile could assist obstetric care providers in initiating timely psychological referrals, delivering targeted interventions, and optimizing prenatal surveillance for high-risk individuals. In an era marked by sustained social turbulence and collective psychological strain, modern obstetrics must move beyond a purely biomedical model. Acknowledging maternal psychological health as a central pillar of prenatal care is no longer optional, it is imperative [24,25,26].

The unexpectedly high rates of adverse outcomes in our cohort warrant comment. First, our sample was drawn from a tertiary university hospital, which often receives more complex cases even among women initially classified as low risk. Second, the study was conducted during the immediate post-pandemic period and ongoing regional instability, factors that likely contributed to elevated maternal psychological distress. These contextual aspects, combined with the cross-sectional design, explain why complication rates exceeded national estimates.

The homogeneity of our cohort strengthens internal validity, as associations between psychological scores and obstetric outcomes are unlikely to be driven by major sociodemographic or medical differences.

Consistent with previous research, the bivariate analysis (Table 4) revealed that elevated scores on the EPDS, GAD-7, and PSS were each significantly associated with preterm birth, gestational hypertension, intrauterine growth restriction (IUGR), and low birth weight [27,28]. Among these psychological variables, perceived stress emerged as the strongest and most consistent correlate—a finding aligned with prior reports suggesting that perceived stress may be a more robust predictor of low birth weight than either anxiety or depression [29].

Although the correlation coefficients observed in this study ranged from small to moderate in strength (r = 0.19–0.36 and r = −0.22), this pattern is consistent with previous reports in perinatal mental health research [28]. Prior meta-analyses have likewise documented modest effect sizes linking depression and anxiety with preterm birth and low birth weight. These relatively weak associations likely reflect the multifactorial etiology of obstetric complications, the transient nature of psychological states during pregnancy, and the impact of contextual factors such as post-pandemic stress and regional instability. Importantly, even weak correlations may be clinically significant, given the high prevalence of psychological distress in pregnancy and the severity of adverse obstetric outcomes [27].

Early identification of pregnant women exhibiting elevated stress or diminished resilience may facilitate timely psychological intervention and more personalized prenatal monitoring. Brief, evidence-based strategies—such as cognitive-behavioral therapy (CBT), mindfulness-based stress reduction (MBSR), or resilience-enhancement protocols tailored to the perinatal context—have demonstrated efficacy in alleviating antenatal anxiety and depressive symptoms. As suggested by our findings, such interventions may also contribute to mitigating the risk of obstetric complications when integrated as adjuncts to conventional obstetric care.

It is also important to situate this study within its socio-political context. Conducted in post-pandemic Romania, against the backdrop of an active military conflict in the region, soaring inflation, governmental instability, and global uncertainty, this research captures a particularly challenging moment for maternal mental health. The elevated prevalence of stress, anxiety, and depressive symptoms observed in our cohort is likely not coincidental, but rather a reflection of the collective psychosocial climate in which these pregnancies unfolded [30,31].

Our findings are broadly consistent with the meta-analysis by Grote et al. (2010) [28], which reported a 40% increased risk of preterm birth among women experiencing antenatal depression. Similarly, the association between trait anxiety and prematurity observed by Rose et al. (2016) [32] was also replicated in our sample. Although no psychological variable emerged as an independent predictor in the multivariate model, perceived stress remained closest to the threshold of statistical significance (*p* = 0.104), reinforcing its potential as a clinically relevant risk marker even when other psychological factors are taken into account [33].

Rather than viewing the lack of statistical significance in the multivariate model as a limitation, we interpret it as a reflection of the complex, multifactorial nature of maternal psychological health. The consistent trends observed in both bivariate and multivariate analyses suggest that perceived stress remains a clinically relevant element, though not the sole determinant, in obstetrical risk. The inverse association with resilience further supports the idea that protective psychological factors, not just vulnerability markers, should be considered in risk assessments.

Resilience, measured by the CD-RISC, emerged as a modest yet consistent protective factor. Its negative correlations with all four complications suggest that adaptive capacity can buffer—not merely moderate—the biological and emotional impact of stress. Women scoring highest on resilience appeared less susceptible to adverse outcomes, even when their stress load was substantial. These findings align with the growing consensus that resilience functions as an independent moderator in the relationship between prenatal stress and perinatal health outcomes [34,35].

The depressive findings align with meta-analytic estimates suggesting that antenatal depression increases the risk of preterm birth by approximately 40%. Regarding anxiety, the GAD-7 yielded a consistent signal: women with higher generalized anxiety scores were more likely to experience preterm delivery or have low birth weight infants—a pattern that mirrors findings from recent studies employing the GAD-7 to assess perinatal risk [36,37].

The findings of this study underscore the importance of adopting a more integrative model of prenatal care—one that transcends purely biological indicators to systematically include maternal psychological health. Instruments such as the PSS and CD-RISC are brief, non-invasive, and readily implementable during routine prenatal consultations. Incorporating them into standard obstetric protocols could yield valuable insights into each patient’s emotional risk profile, especially in settings characterized by widespread social stress or instability.

The multivariable logistic regression model (Table 5) further clarified these patterns. Among all psychological predictors, only GAD-7 scores retained a statistically significant association with the composite obstetric outcome after mutual adjustment. Perceived stress approached significance, while depressive symptoms and resilience lost their predictive value once shared variance was controlled. This configuration underscores the disproportionate influence of antenatal generalized anxiety on obstetric risk, even amid overlapping mood-related constructs.

This knowledge gap is especially relevant in the current global and regional context. The present study was conducted in post-pandemic Romania, a country facing a particularly challenging socio-political climate [38]. Although not directly involved in military conflict, Romania shares a border with Ukraine and lies in close proximity to ongoing war zones. The prolonged regional instability, persistent media coverage of conflict, and fear of escalation have contributed to a sense of uncertainty and existential threat among the population [39]. Added to this is a recent sequence of domestic political crises—including annulled elections, the temporary absence of a functioning government, and widespread distrust in public institutions. These are compounded by record inflation, economic strain, and mounting global concerns such as climate change, resource insecurity, and even the looming discourse around a potential third World War. Together, these overlapping stressors contribute to a psychological climate in which pregnancy-related vulnerabilities may be amplified rather than mitigated.

This study aimed to explore the relationship between key maternal psychological factors, depression, anxiety, perceived stress, and resilience, and the occurrence of obstetrical complications in a sample of pregnant women in post-pandemic Romania. The findings align with the growing body of international literature suggesting that maternal psychological vulnerability can significantly influence pregnancy outcomes, particularly in times of social instability. Although the peak of the COVID-19 pandemic has passed, a lingering sense of uncertainty and concern about potential future pandemics continues to permeate public consciousness, contributing to a sustained atmosphere of psychological tension and anxiety [24,25,26].

This study offers a valuable contribution by integrating validated psychometric instruments with systematically documented obstetric outcomes. The results reinforce the rationale for incorporating psychological screening—particularly for perceived stress and resilience—into standard prenatal protocols. Although the multivariate analysis did not identify statistically independent predictors, the strength of the bivariate associations and the clinical salience of observed trends underscore the potential value of establishing structured psychological risk profiles during pregnancy. Such profiles may serve as important tools for anticipatory guidance and targeted prevention, especially in times of collective adversity and uncertainty [40].

Ultimately, our results reinforce the need for standardized prenatal psychological screening, at a minimum for perceived stress and resilience. Even in the absence of statistically significant predictors in multivariate analysis, the robustness of the bivariate relationships and the clinical implications justify early preventive interventions. Identifying pregnant individuals with high PSS scores or low resilience could guide personalized monitoring and targeted psychological support throughout the pregnancy [41].

### Future Directions, Strengths, and Limitations

This study opens promising avenues for further research into the integration of psychological assessment within standard prenatal care. Future studies should aim to longitudinally track the psychological trajectories of pregnant women from early gestation to postpartum, in order to better understand the dynamic relationship between stress, resilience, and obstetrical outcomes. Moreover, the development and validation of a standardized psychological risk screening tool, adaptable to diverse clinical settings, would significantly enhance early identification and intervention strategies. Incorporating biological correlates, such as cortisol levels or inflammatory markers, alongside psychological data may offer a more nuanced understanding of the biopsychosocial mechanisms underlying adverse pregnancy outcomes. Intervention-based studies evaluating the impact of targeted psychological support on both maternal well-being and perinatal outcomes are also warranted.

One of the main strengths of this study lies in its real-world clinical setting within a tertiary-level university hospital, which ensures the relevance and applicability of findings to everyday obstetrical practice. The inclusion of a geographically and socioeconomically diverse patient population enhances the generalizability of the results across different subgroups of pregnant women. Additionally, the use of validated and widely recognized psychometric instruments (EPDS, GAD-7, PSS, CD-RISC) provides methodological rigor and comparability with the international literature.

Several limitations should be acknowledged. First, the cross-sectional design restricts causal inferences between psychological factors and obstetrical outcomes. Second, self-reported measures may be subject to recall bias or social desirability bias, potentially influencing the accuracy of responses. The lack of long-term follow-up limits insight into how psychological status evolves over time or impacts postpartum health. Additionally, certain psychosocial variables such as trauma history, quality of intimate relationships, or cultural beliefs were not assessed, which may also play a significant role in shaping maternal psychological resilience and perceived stress. Given the cross-sectional design, causality cannot be inferred. Psychological distress should be considered as a vulnerability marker rather than a direct cause of adverse outcomes. Although none of the participants had a documented active COVID-19 infection in the three years preceding conception, prior mild or asymptomatic infections could not be systematically excluded and may represent a source of residual confounding.

## 5. Conclusions

This study found that elevated levels of perceived stress, anxiety, and depressive symptoms were significantly associated with adverse obstetric outcomes, including preterm birth, gestational hypertension, intrauterine growth restriction (IUGR), and low birth weight. Of all psychological variables assessed, perceived stress demonstrated the strongest and most consistent associations. Although no predictor retained statistical significance in the multivariate model, perceived stress approached the threshold (*p* = 0.104), underscoring its potential clinical relevance.

Conversely, resilience was inversely associated with all obstetric complications, indicating a potential protective role. Women with higher resilience scores appeared less susceptible to adverse outcomes, even in the presence of elevated stress levels, underscoring the moderating impact of psychological adaptability.

In light of the current socio-political climate in post-pandemic Romania, these findings underscore the importance of incorporating psychological assessments into routine prenatal care. Brief and easily administrable tools such as the PSS and CD-RISC could serve as valuable instruments for early identification of women requiring targeted psychological support.

The present findings support the formulation of a structured psychological risk profile for pregnancy. Despite the absence of statistically robust multivariate predictors, the consistency of the bivariate associations underscores the clinical rationale for early screening and tailored interventions to enhance maternal and fetal outcomes.

## Figures and Tables

**Table 1:** Demographic characteristics of the patients.

Variable	Value
N	240
Age (years)	30.22 ± 4.01
Gestational age at the time of enrollment (weeks)	24.59 ± 2.11
Gestational age at birth (weeks)	39 ± 1.18
BMI	25.74 ± 3.75
Higher education (%)	152 (63.33%)
Secondary education (%)	88 (36.66%)
Urban residence (%)	176 (73.33%)
Rural residence (%)	64 (26.66%)
Married (%)	208 (86.66%)
Stable partnership (%)	32 (13.33%)

**Table 2:** Descriptive statistics of psychological scores.

Questionnaire	Scale Range	Mean	Standard Deviation	Patients Above Clinical Cut-Off
EPDS Score (0–30)	0–30	11.03	5.25	92.0
GAD-7	0–21	11.0	4.3	120.0
Perceived Stress Scale—PSS (0–40)	0–40	20.52	5.61	139.0
Connor–Davidson Resilience Scale—CD-RISC (0–100)	0–100	60.78	14.76	118.0

**Table 3:** Obstetric complications.

Complication	Number of Cases	Percentage (%)
Preterm Birth (<37 weeks)	51	21.25
Gestational Hypertension	41	17.08
Intrauterine Growth Restriction (IUGR)	36	15.0
Low Birth Weight (<2500 g)	50	20.83

**Table 4:** Correlations between psychological scores and obstetric complications.

Predictor	Complication	Correlation Coefficient (r)	*p*-Value
EPDS	Preterm Birth	0.28	0.001
	Gestational Hypertension	0.24	0.004
	IUGR	0.19	0.018
	Low Birth Weight	0.26	0.003
GAD-7	Preterm Birth	0.33	0.0003
	Gestational Hypertension	0.28	0.0019
	IUGR	0.23	0.011
	Low Birth Weight	0.31	0.0008
PSS	Preterm Birth	0.36	0.0001
	Gestational Hypertension	0.32	0.0007
	IUGR	0.28	0.002
	Low Birth Weight	0.33	0.0003
CD-RISC	Preterm Birth	−0.29	0.002
	Gestational Hypertension	−0.25	0.008
	IUGR	−0.22	0.012
	Low Birth Weight	−0.27	0.004

**Table 5:** Multivariate logistic regression.

Predictor	Coefficient	Standard Error	z	*p*-Value	CI Lower	CI Upper
const	−1.548	1.622	−0.954	0.34	−4.728	1.632
EPDS	−0.001	0.033	−0.021	0.983	−0.066	0.065
GAD-7	0.047	0.019	2.470	0.013	0.010	0.083
PSS	0.051	0.031	1.627	0.104	−0.01	0.112
CD_RISC	−0.003	0.011	−0.288	0.773	−0.024	0.018

## Data Availability

Data are available upon request from the corresponding author.
